# COVID-19: Chittagong Port and aftermath

**DOI:** 10.1007/s13437-021-00234-2

**Published:** 2021-05-31

**Authors:** Samsul Mannan, Md. Mostafa Aziz Shaheen, Rana Saha

**Affiliations:** 1 Marine Department, Chittagong Port Authority, Chittagong, Bangladesh; 2 Department of Port and shipping management, Bangabandhu Sheikh Mujibur Rahman Maritime University, Dhaka, Bangladesh; 3 Department of Maritime Operations, University of South-Eastern Norway, Vestfold, Norway

**Keywords:** Impact of COVID-19, Chittagong Port, Port and shipping, Bangladesh

## Abstract

After first unveiled in China, by March 2020, the wide spreading episode of coronavirus diseases 2019 (COVID-19) spread over Europe and the rest of the world. Besides the public health crisis, not only the Chinese economy but also the global economy as well as the supply chain faced a significant slowdown. Port is not apart from this. Chittagong Port is considered the economic nerve of Bangladesh. A significant segment of export-import moved through Chittagong Port. Like the entire globe, the outbreak of coronavirus affects the country’s prime seaport adversely. This study examines the issues concerning port operation. This study has a qualitative approach that depended on data collected from both primary and secondary sources. Primary data were collected from field surveys using semi-structured questionnaires and in-depth interviews with government agencies, policymakers, researchers, businessmen, port users and entrepreneurs. Secondary data were sourced from different policy documents, scholarly articles, reports and the internet. This study provides an overview of COVID-19 impacts on the port and shipping activities in Chittagong Port, which include impacts on port of call, warehouse and distribution activities, hinterland connections and port employees. Therefore, this study discussed those issues to date, identify impacts and authority response to address the impacts. Finally, this study put forward strategies for their mitigation.

## Introduction

The coronavirus diseases (COVID-19) were unveiled in a Chinese transport hub, Wuhan, on 31 December 2019 (World Health Organization 2020). China involves a global share of 16% GDP (29% manufacturing, 13% export and 18% manufactured exports) and makes the country a major supplier of raw materials (Baldwin and Weder di Mauro 2020). Lockdown in Wuhan due to COVID-19 affects the manufacturing activities across the globe (Baldwin and Weder di Mauro 2020). By March 2020, the virus spread over Europe and the rest of the world. Besides the public health crisis, the global economy as well as the supply chain faced a significant slowdown (UNCTAD 2020). Due to economic disruption generated by the COVID-19 pandemic, global trade is expected to fall by 13 to 33% in the current year forecasted by a report of the World Trade Organisation (Jackson et al. 2020).

Port is a key component of the supply chain. Port acts as an intermediate point to transfer goods from manufacturer to customer. Whether goods are raw materials or finished goods, essential or luxury. Every 10% growth of a port throughput can produce a 6–20% increase in the GDP of that region (Bottasso et al. 2013). A fall in trade flow reduces the port and shipping growth. As the principal seaport, Chittagong port is recognized as the nerve of the economy of Bangladesh. More than 90% of the nation’s trade performed through this port (Mahmud and Rossette 2007). Chittagong Port Authority (CPA) achieved a millstone of handling more than 3 million TEU’s (Twenty Equivalent Unit) last year. Like the entire globe, the outbreak of coronavirus affects negatively the country’s prime seaport. Export-import through Chittagong Port experienced a decline from January 2020. In April, CPA faced a noticeable fall of export-import by 46.76% in containers (TEU) and 27.59% in bulk cargo (MT) (CPA 2020). The unprecedented COVID-19 pandemic has displayed vulnerability in maritime networks, port efficiency and hinterland connectivity.

This study aims to discuss those issues to date and intends to provide an overview of COVID-19 impacts on the port and shipping activities in Chittagong Port. This study also discussed the response by governments to address these effects respective to the Chittagong Port. Moreover, in this study, various perspectives related to port operation including the port of call, waiting period and turnaround time are identified from the subject experts, which will accelerate efficient planning and organization of port activities. Regional and local port authorities, port users and decision-makers may consider these perspectives for their future preparedness.

## Background

Since this is a unique and novel situation, til to date, only a few works have covered the impact of the port operation sector. International Association of Ports and Harbors (IAPH) and its World Ports Sustainability Program (WPSP) jointly surveyed COVID-19 impacts on port economics by barometer survey. In this study, the researcher primarily revised the IAPH-WPSP COVID-19 impact barometer by Professor *Theo Notteboom* and Professor *Thanos Pallis*. Besides, the researchers also analysed the UNCTAD (United Nations Conference on Trade and Development) report on this issue. Initially, the half-yearly report of the IAPH-WPSP COVID-19 impact barometer conducted a survey based on the theme of vessel calls, extra restrictions on vessels, port call procedures, hinterland transport, warehousing and distribution activities and port-related workers. These six criteria have direct impacts on the development of COVID-19 in the port and shipping industry. However, these criteria are also interconnected with each other as all of these are part of the same logistic platform. Since the first three of these themes are related to ‘port of call’, this study merged them into one. Eventually, this study was conducted based on the following four themes—impacts on port of call, hinterland transport, warehousing and distribution activities and port employees.

### Port of call

According to the dictionary by Merriam-Webster, a port of call is ‘an intermediate port where ships customarily stop for supplies, repairs or transhipment of cargo’. While vessel calls at the port, port users are concerned with waiting time, meaning that select a port where lower waiting time. Kavirathna et al. (2018) defined waiting time as the time that vessels have to wait at the anchorage area before the port entrance and turnaround time as the total time a vessel stays in port for its entire operation.

According to the IAPH-WPSP impact barometer, several factors lessened the vessel’s call at the port of different regions worldwide. Due to cargo volume shrinkage, container liner itinerary postponing experienced by ports. Besides, some ports were adversely affected due to the closing of Asian and European destination ports. The government imposed restrictions and decleared an emergency state that discontinued the economic activities of several general cargo ports worldwide. Except for the essential commodities, no bulk vessels carrying construction cargo called at the port of different regions as construction work stopped at lockdown period. Besides, due to the closing of terminals and travel restrictions, cruise ships stopped their operations and laid off their vessels at berth with a limited crew on-board (Notteboom and Pallis 2020).

To combat the COVID-19, several protocols were introduced by ports worldwide. Sanitization certificates are made obligatory in most cases. COVID-19 protocols involved health declarations for both vessel crews and ground staffs. These protocols also included no landing permit and sanitary inspections that applicable to vessel crews. Besides, maintaining social distances, using basic PPE (personal protective equipment) facemask, goggles and hand gloves made mandatory combating COVID-19. However, some ports permit crew transfer on a limited scale. Besides, 14 days of quarantine periods with tests imposed on suspected vessels in many cases (Notteboom and Pallis 2020).

**Fig. 1:**
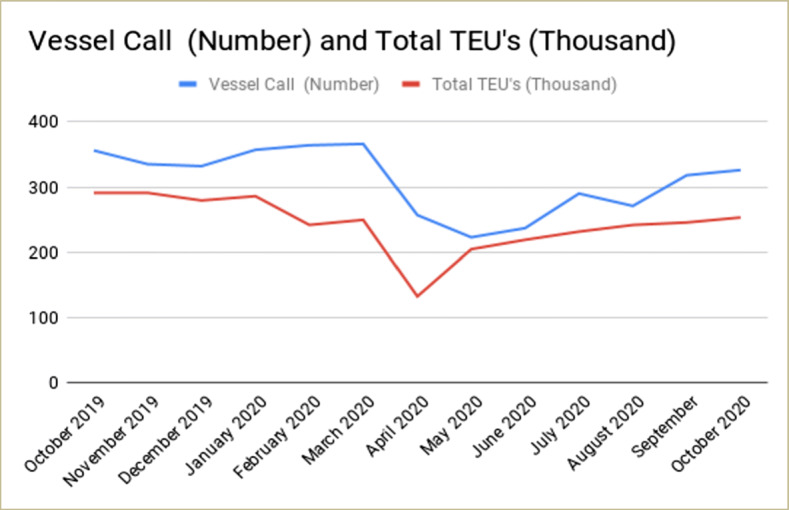
Number of vessel calls and cargo volume

The Department of Shipping, Bangladesh, issued several circulars for seaport and ships regarding coronavirus. According to these guidelines, Chittagong Port imposed a 14-day mandatory quarantine period as well as designated quarantine anchorage for the vessels trading between mainland China and Bangladesh directly or via intermediate ports. As a result, vessels had to experience significant waiting periods for berthing and long turnaround time than the average at Chittagong Port. Also, vessels calling Chittagong Port have to submit mandatory health and medical declaration forms in addition to mandatory COVID-19 health protocol. On the other hand, the number of vessel calls fell significantly compared to a normal situation due to cargo volume reduction (Shamsuddin Illius 2020). Figure 1 shows the number of vessel call and cargo volume (in thousand TEUs) between October 2019 and October 2020.

### Warehouse and distribution activities

In recent days, warehouse and distribution activities shifted from a simple transport procedure to an integrated system based on large distribution centres which include large buildings with storage, cross-docking, customization, light processing (minor modification/final adjustment) and information management (Monios et al. 2018). With the shifting of ports to outer city areas, much of the distribution activities have moved inland which resulted in less congestion in the port areas (Monios et al. 2018). Chittagong Port also has had a similar progression in recent days with 19 Inland Container Depot (ICD) out of port areas.

Full or partial lockdown in many nations affects not only consumer demand but also in the industries. Consumer demand fell as well as industries were closed. The businessmen practised hoarding in many cases. Oil prices sharply declined globally, as there was less demand. Consequently, storing oils by the liquid bulk trader for future price development resulted in a shortage of storage capacity. Essential bulk goods such as grain (rice, wheat) experienced the same. Contrary, non-essential bulk cargo export was close to zero. Due to the sales collapse, the automobile market faced an extremely critical situation. The warehouse became full as the traders failed to release their new cars.

According to *CPA Container Manual*, in Chittagong Port, container cargoes are shipped or released directly through port or ICD for staffing, un-staffing, storage and customs procedure. Due to the closure of industry during the lockdown period, import cargo unloading became at a standstill. Besides, limited custom activities and transport shortage cause a record of container stock at the port yard (Shamsuddin Illius 2020). In response to this situation, the National Board of Revenue (NBR) issued an official order allowing direct transfer of shipments to private ICDs from fixed 38 cargoes to all products until June 30 (The Daily Star 2020).

### Hinterland connection

Jeroen de Haas defines port hinterland as a backhand area of the port, served by that port with an extensive network of road, rail or barge connection. As a gateway of export-import, the port supports a large hinterland (Jeroen de Haas 2016). Veenstra et al. (2012) addressed that better performance of the supply chain can be achieved by transport network integration from the sea terminal to the hinterland. Moreover, the accessibility and performance of inland link are considered as one of the vital standards in selecting deep seaport by the container shipping lines (Wiegmans et al. 2008).

The IAPH-WPSP report identified bottlenecks that disrupt the hinterland as well as port supply chain during the COVID-19 crisis. Cross border restriction, unavailability of the trucker and terminal operation disturbance affected adversely on port hinterland operation. In some cases, trucking operations, rail and inland water services had been halted due to government-imposed restrictions resulting in congestion at the yard. Due to space scarcity, downstream warehouses could not accommodate the yard (Notteboom and Pallis 2020).

The hinterland connection of the CPA is a mix of road, rail and inland waterways (mostly depend on road transportation). The capital city, Dhaka, is the main hinterland for the Chittagong Port. Close to 70% of the consignment handled at this port is originated and destined to the Dhaka region (Hasan and Khondoker 2016). Preventing the spread of COVID-19, the government announced a general holiday from 26 of March to 11 of April this year. The importers could not ship the consignments due to the scarcity of vehicles. Approximately 5000 transport vehicles typically enter the port for deliveries and shipments daily. The number of vehicles reduced to 800–1000 as the drivers were not keen to drive in fear of the COVID-19. The operation of customs and banking activities ran on a limited scale resulting in stuck-up cargo at the port (Shamsuddin Illius 2020).

### Port employee

Hooydonk defines port workers as a pool of workers that is considered as a low-skilled manual profession (Van Hooydonk 2014). Barton and Turnbull stated port/dock workers serving within the port zone under the joint management of the port employers and the unions (Barton and Turnbull 2002). However, according to the CPA ordinance 1976 (section 52), port employees include all permanent and temporary employees employed under CPA.

IAPH-WPSP report stated that one-third of the ports experienced a shortage of personnel once the government imposed lockdown and other associated rules to confront the outbreak of COVID-19. This unavailability was more on marine department which mostly engaged on pilotage, towage and berthing services. To avoid departmental quarantine, shift work practice was adopted by the port authorities. Port employees maintained social distancing and protective safety measures to prevent ‘person to person’ transmission of COVID-19.

According to CPA Annual Report 2017–2018 (published on 26/08/2019), 8679 port employees under 17 departments including marine, mechanical, traffic, security, electricity, hospital, etc. are working for CPA. Nearly 20 organizations (CPA annual report 2017–2018) are directly involved with the port operation where about 30,000 people directly work inside the port area per day (Monir 2017). As the busiest seaport of Bangladesh, CPA was operational round the clock during the government-imposed COVID-19 restriction period (Hussain 2020). Port employees were recognized as ‘emergency workers’ to combat COVID-19 to attain priority access on movement during the lockdown period. During this period, CPA faced a shortage of personnel. To ensure operational smoothness, CPA introduced work shifts in a format of ‘one week on and two weeks off’. Besides, the port authority provided safety protective materials to keep workers safe. Moreover, within its premise, CPA placed a COVID-19 test booth and established a 70 bed COVID-19 ward within its hospital with a high flow oxygen supply facility. As of August 2020, COVID-19 incentive and medical expenses for CPA employees are under process.

## Methodology

This study is qualitative research based on both syntheses of secondary information and primary data. *Qualitative research* is a process of naturalistic inquiry that seeks an in-depth understanding of social phenomena within their natural setting and seeks to justify underlying reasons, opinions and motivations (Drury, Homewood, & Randall, 2011). To collect secondary data, different policy documents and scholarly articles related to COVID 19 impacts, and various government websites, were reviewed. Besides, data from various government documents were consulted, which include various circulars about COVID-19 from Chittagong Port, shipping ministry and other departments. Subsequently, to collect primary data, a total of 13 semi-structured interviews were conducted from six (06) different informants categories, i.e. government officials, policymakers, researchers, businessmen, port users and entrepreneurs. A detailed overview of the interview and respondents is given on the Appendices.

Analysis of the primary data is developed following the thematic analysis method. Thematic analysis is *a method for capturing patterns* (‘themes’) *across qualitative datasets* (Braun et al. 2018). In semi-structured interviews, a standard set of guiding questions were used. Nevertheless, when an interesting or new line of inquiry developed during the interview process, additional questions about the topic were asked. Thus, the semi-structured interview technique made it possible to go in-depth about a particular topic. It also helps the interviewer to ‘probe’ for obtaining the details from several perspectives. At the same time, a semi-structured interview provides the informants with the freedom to speak in a broad and wider context. After transcribing the interviews, coding of the interview transcript was done. Coding is a process for identifying interesting and silent feature of the text within the text that relates to the research question or research objectives (St. John et al. 2014). According to the research objectives, many codes were created for every interview transcript. Then, the codes, which relates to similar research objectives, were grouped together to form an overarching theme that was made according to the research question. According to the research question, four themes were identified, which include impacts of COVID-19 on vessel call, warehouse and distribution activities, hinterland transport system and port employee.

**Fig. 2:**
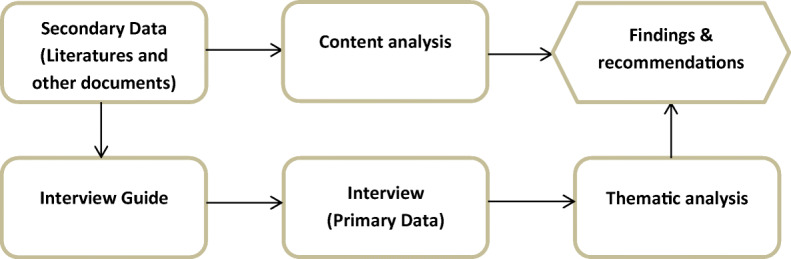
Research method

For secondary data analysis, the content analysis method was used, which is a research tool for interpreting and understanding the inner meaning of the textual material, articles and graphics (Shamsuzzaman & Islam, 2018). The content analysis method helped to understand the inner meaning, i.e. the gist of various policy documents, government circulars and reports. Since both the thematic and content analysis methods are having a qualitative approach to obtain the pattern/theme/inner meaning from the text/content, both the method helped to get findings and recommendations in a qualitative way, which is discussed later on in section 05. Figure 2 provides an overview of the research method of this study.

Similar to any other study, this study also has some limitations. This study was conducted during a crisis period while a majority of the people was impacted by the crisis. Reaching out to various categories of informants was the primary challenge for this study. However, there is only one informant in three categories. Again, having a good number of information’s from six different categories who are closely related within their service area can compensate for this lacking. The impact of the COVID-19 crisis is not yet over. The cumulative impact from this pandemic shall be different from the impacts shown at the current stage.

## Interview analysis

To have an idea of the overall impacts of COVID-19, interviewees were asked about the overall performance of the port during the COVID-19 period. While answering this question, different interviewees answered in different ways. For example, according to high officials from the marine department—the port performance did not drop significantly as no berth was empty during the COVID-19 lockdown period (general holidays announced by govt. of Bangladesh). He argued that there was always used to be a queue of 12–13 ships regularly in the port. He also added that due to the increase of scrap imports, many ships used to wait for getting berths. Thus, whenever there was a chance, those scrap-carrying ships took the berth and made the berth occupancy more than 90% during COVID-19 lockdown days. On the other hand, officials from the traffic department and shipping agents argued that port performance was not up to the mark due to severe congestion of containers inside the port. The traffic department mentioned that, on the 24th of April, against the capacity of 32,000 full container load (FCL) containers, Chittagong Port had 42,000 FCL containers stored in the port premises, which means the stock was 32% over the capacity of the port. This high congestion of containers dropped the performance of the port. Subsequently, the planning department had a different view on the overall impact of COVID-19 in the Chittagong Port Authority (CPA). According to the planning department, the impact of COVID-19 on Chittagong Port, especially in the development sector, is enormous. High officials from the planning department stated that CPA is conducting several development projects, including Matarbari Port development Project, Constructing Potenga container terminal (PCT) and Bay Terminal, which was affected tremendously due to COVID-19. Among these development projects, Matarbari Port is the government’s fast track projects which are being implemented under the financial assistance of the Japan International Cooperation Agency (JICA). CPA has already completed the negotiations with the consultants in early October 2019, but CPA could not sign the contract agreement with the consultant because of COVID-19. The signing of the agreement was supposed to be completed in April 2000. Thus, all the processes of the Matarbari project were impaired because of COVID-19. As most of the foreigners including the JICA people have left the country, every stakeholder is now in a dilemma as to when CPA will be able to sign the contracts. Besides, the return of foreigners is uncertain which will surely delay the achievement of the planned schedule of these projects. Other development projects like capital dredging, overflow yard and all other project implementation are also on hold leading to a huge financial loss for CPA. Furthermore, official from the port, training institute tried to relate the statistics of export-import with port performance. According to him, as the export has reduced 9% during the coronavirus period, the port performance has also reduced.

After gathering relevant information from respondents, it is abundantly clear that COVID-19 impacted port performance tremendously. Thus, to better understand the impacts on the port due to COVID-19, the researcher tried to summarize the impacts which include impacts on vessel call, waiting period, turnaround time, warehouse, ICDs & Distribution activities, hinterland transport and port workers and find out the core reasons behind these impacts.

### Impacts on port of call

When interviewees were asked about the impacts on vessel call, different opinions came from different interviewees. For example, officials from *VTMIS* (Vessel Traffic Management Information System) emphasized the impacts on vessel call procedure. He stated that due to COVID-19, the vessel was required to go through extra precautionary procedures which include providing information about the ‘Pre-Arrival Health Declaration form’. This form was specially created for the COVID-19 period. According to this form, vessels needed to provide various information like name and flag of the ship, number of crews in the ship, nationality of crews, last five ports of call with dates and departure date of last port. If there were any Chinese ports within the last five ports of call, then vessels are required to provide more information which includes information about crew nationality and crew illness. Besides, only to handle COVID-19 related documents, CPA opened an email address (mcov2019@gmail.com). All the coronavirus-related documents and information were exchanged through that email address. Moreover, the vessel whose last port was a Chinese Port had to wait to get a berth till she finished her mandatory quarantine period (14 days). Since a ship directly coming from China to Chittagong Port requires a 10-day transit time, the ship had to wait more than 4 days to complete the mandatory quarantine period to get a berth in the port. After completing the quarantine period, a port health officer was required to board the ship to check the physical condition of the ship’s crew. After obtaining the quarantine certificate, the vessel was eligible to get the berth. Most of the interviewees agreed that this process slowed down the vessel call procedure.

After getting a clear picture of the vessel call procedure, interviewees were asked about the impacts on the vessel’s number, turnaround time and the waiting period of the ship. When the questions were asked about the impacts of COVID-19 on vessel calls, almost all the interviewees agreed that vessel numbers were drastically reduced during the coronavirus period. According to officials from the marine department, the number of container ships coming to port decreased to 60 ships from 80 ships in a month.

When the question was asked about the reason behind the decreased vessel number, interviewees tabled different opinions including reduced banking hour, limited scale custom facility and closing of garments. According to the traffic department, reduced banking hours was the basic reason, which impacted tremendously on the vessel call. In addition, a representative from the ship agents association included that customs were working on a limited scale with limited human resources. Thus, the importer could not take the delivery of their containers from the port, which made the ship stay in the port for extra days. Moreover, a representative from the shipping agent association mentioned that majority of the export in Bangladesh is from the garment sectors and all the garments were closed during the lockdown period. Thus, the delivery of containers from the port was near to the ground. Consequently, the port capacity was exhausted and impacted the vessel call.

Obtaining information about the impacts on vessel calls, the interviewees were asked about the impacts on the waiting period and turnaround time. Most of the interviewees agreed that the vessel’s waiting period for berthing and turnaround time was longer than before due to COVID-19. According to the traffic department officials, on the 24th of April 2020, the number of ships on outer anchorage waiting to take berth were increased to 37, which was the highest in Chittagong Port history. Simultaneously, the waiting period to get a berth for a vessel also increased up to 10 days. Allowing direct delivery from the off docks on the 30th of April, the number of vessels waiting was reduced to eight (08) and the number of waiting days was reduced to 4–5 days in the outer anchorage. As the situation improved, after the 30th of June, the number of waiting vessels in the outer anchorage was four (04) and the number of waiting periods to get berths was almost 2–3 days.

When the question was asked about the reason behind the increased waiting period, different stakeholders argued with different opinions. According to the officials from the marine and traffic department, three reasons that caused the waiting period to increase suddenly. Firstly, the unwillingness of importers to take the delivery of containers includes fresh fruit containers and cars. They intended to use the port as their storage and take delivery as per demand and sell in the retail market. On the other hand, according to the representative from the ship agents association, the sudden onset of a global pandemic that had no warning signs made the trade community unprepared and bound them to keep their containers in the port causing huge congestion. The second reason was the slow discharge rate of containers resulting in congestion of containers in the port premises. As per the traffic department, on the 24th of April, a total of 90,000 containers were piled up which were not being delivered to their destination. Before pandemic, CPA used to discharge 4000 containers daily, which came down to 1000 containers per day during the initial pandemic period. Thus, the vessels at berth could not sail in time, which increased the turnaround time and waiting period. Thirdly, delay in the decision-making to decongest the port. Due to coronavirus, the port exempted the storage rent for the goods for certain periods. Thus, the businessman took advantage and did not take delivery of their container. Besides, the importers wanted the government to give them an incentive package as well as a 100% tax exemption on the imports. So, they were reluctant in exporting before government decisions on it. They wanted this package and tax exemption before they export. Ultimately, tax exemption was not given and it took a long time to make the decision, which impacted the waiting period negatively.

Moreover, the representative from the ship agent’s association mentioned that the 14-day mandatory quarantine protocol imposed by the CPA increased the vessel waiting period. He argued that maximum import vessels are coming from China and according to the quarantine protocol, any ship coming from China had to wait at outer anchorage for 14 days, which increased the waiting period. Besides, officials from the CPA training institute pointed out that CPA does not have sufficient jetties. Thus, the lack of jetties is another perennial reason which increased the waiting period.

Therefore, to understand these aforementioned issues, based on the interview data, the researchers divided the total time frame into three (03) phases and prepared a table showing all the impacts on vessel call:

- 1st phase, 26th of March to 24th of April: As the public holiday/general leave/lockdown started in Bangladesh on the 26th of March and restriction started, the time frame of the first phase is from the 26th of March to the 24th of April.
- 2nd phase, 24th of April to 30th of June: As the waiting period of the vessel was increasing, CPA, Ministry of Shipping and other stakeholders started taking action on the 24th of April. Then, the waiting period started to reduce. Thus, the second phase started on the 24th of April and finishes on the 30th of June.
- 3rd phase, 30th June-onwards: After the 30th of June, the waiting period came to normal. Thus, the third phase includes the 30th of June onward time.

Table 1 explained it further.

**Table 1:** Impacts on vessel call (based on data from CPA)

Different phases	Time frame	Vessels waiting to take berth	Vessel waiting period	Container stored (approximate)
1st phase	26th of March to 24th of April	37	9–10 days	42,000 FCL containers
2nd phase	24th of April to 30th of June	8	4–5 days	36,000 FCL containers
3rd phase	30th of June onwards	4	2–3 days	34,000 FCL containers

After gathering information about impacts on vessel calls, interviewees were asked about the measures taken by CPA. In response to this question, officials from the marine department mentioned the meeting conducted with trade bodies to decongest the port. He also added that, on consensus with the trade bodies, CPA decided to avail the empty spaces of the off docks. Consequently, some space was created in the port yard, which helped to mitigate the congestion in 25–26 days. Besides, according to the traffic department, Chittagong Port waived the landing charge of the container in 3 steps which were more than fifty billion takas (58.82 million USD). This waiving of the container landing charge encouraged the importers to take the containers to their premises. Moreover, according to the representative from shipping agents, direct delivery of the containers to the off docks reduced the waiting period of the vessels.

### Impacts on warehouse and distribution activities

When the questions were asked about the impacts on the warehouse and distribution activities, most of the interviewees agreed that COVID-19 had tremendous impacts on the warehouse and distribution activities. In this issue, representatives of the shipping agents mentioned that there was huge congestion of reefer containers in the reefer yard as the importers were not taking delivery of the apple containers. In the sudden onset of the pandemic, the demand for the apple was reduced and the demand for the orange was increased. Therefore, the apple containers were piled up in the port premises. Thus, the number of reefer containers was more than the capacity. For this reason, to keep the quality of the reefer container, port officials had to make rationing of the power supply, thus each reefer container was used to get a plugin of 2 h of power supply in alteration.

Due to COVID-19, importers had to use private ICDs. About this issue, officials from the CPA admin department admitted that the delivery process from these ICDs was cumbersome and costly, as the importer had to pay a higher charge than the Chittagong Port for taking delivery of a container. When the question was asked about the activity of the ICDs and port facility, most of the interviewees agreed that private ICD’s helped a lot to decongest the port and mentioned to increase the warehouse capacity of the port facility. Thus, the question was asked about increasing the warehouse capacity of the port facility. In this issue, officials from the planning department mentioned the development of the new terminals including bay terminal, Potenga container terminals and Matarbari Port which are slowed down due to COVID-19. Moreover, he mentioned that Chittagong Port is the only port where all the container freight station (CFS) sheds are inside the port which means that all the stuffing and de-stuffing of FCL containers are performed inside the port. During the pandemic, this CFS shed made the port more congested. Thus, the question was asked about the shifting of these CFS sheds. In this issue, officials from the planning department answered that CPA has proposed a CFS shed in the bay terminal area. But unfortunately, this proposal was not accepted by the ministry due to bureaucratic bottlenecks, i.e. blockades from other ministries. Therefore, the interviewees were asked about constructing a new CFSs shed and delivery yard outside the port area; most of the interviewees agreed that CPA must construct a new delivery yard and CFS shed outside the port area. Officials from the planning department think that CPA should construct a new delivery yard outside the port area with flyover and rail connection so that CPA can distribute the loose cargo to this yard without interrupting the city traffic. He also added that due to COVID-19, there was huge congestion in the Dhaka ICD. Thus, no wagon was leaving from Dhaka, which created a wagon shortage in the Chittagong Port. This wagon shortage made more congestion in the port premises.

### Impacts on hinterland connectivity

According to most of the interviewees, the impact of COVID-19 on hinterland connectivity was tremendous. A representative from the marine department mentioned that, due to lockdown, initially there was an acute shortage of transport workers including drivers and helpers. This sudden onset of the pandemic made the people afraid and panicked. Thus, the transport workers were unwilling to perform their duty. But, when the government declared the transport of goods as an essential service, then the situation improved. On the other hand, officials from the traffic and planning department had different views. As per the representative from the planning department, due to the pandemic, people were at home and the traffic was less. Thus, it took more time to deliver cargoes to the warehouse. Whereas official from the traffic department emphasized on rail connectivity. He thinks that the Chittagong Port suffered from huge congestion due to the wagon shortage, as no wagon was leaving Dhaka because of congestion in Dhaka ICD. However, later on, CPA reduced this congestion by transferring these containers to the private ICD’s. On the other hand, a representative from the shipping agent pointed out the distance location of private ICD’s. He tabled that the location of private ICD’s is far away from the port and the road connectivity is not good. Moreover, due to the pandemic, regular maintenance of the road was interrupted. As a result, the hinterland connectivity between the port and warehouse became worse. Thus, due to the distanced location and road connectivity, it was difficult to bring containers to the hook point from those ICD’s.

When the question was asked about what should be done to improve hinterland connectivity, officials from the training department mentioned that the CPA should extend the port link road from four lanes to eight lanes. He also added that transport workers should be provided with PPEs and incentives as well as training on social distancing. Besides, a representative from the ship agent association emphasized constructing new flyovers to connect the private ICD’s with the port. On the other hand, officials from the planning department emphasized improving rail connectivity.

After gathering all the interview data, it became eminent that initially, there were tremendous negative impacts on the hinterland connectivity. But after the government declaration about the transport of goods as an essential service, the negative impact reduced.

### Impacts on port employees and workers

When the question asked about the impacts on the port employees and workers, most of the interviewees tabled that COVID-19 had tremendous impacts on the port workers. According to the medical department, till the 30th of July 2020, almost 500 people were affected by the COVID-19 and 40 people died due to COVID-19. Thus, the question was asked about measures taken by the CPA to fight against COVID-19.

According to the marine department, the measures taken by CPA were sufficient. Officials from VTMIS added that during pandemic CPA reduced human gathering in the port premises. CPA reduced the paperwork by the implementation of submitting the online document. All the bills including water bills, tug bills, pilot booking, cancellation and letter from ships were received and distributed via email. According to the traffic department, the reduction of the human gathering was introduced by distributing the workforce into three groups. In the traffic department, the 1st group worked for 1 week and went for quarantine, then the second group worked in the second week, after the second week, the third group worked in the third week and in the fourth week, the first group came back to work after quarantine. By introducing this rotation and rostering system, the traffic department reduced the impact of COVID-19 and kept the port operational. On the contrary, representatives from the ship agent’s association argued that this rotation system reduced the efficiency of the port. Besides, the marine department mentioned that, to protect operational people from infection, they provided personal protective equipment (PPE), sanitizer, gloves and goggles. Moreover, people were instructed to maintain social distancing. According to the medical department, CPA started an Isolation Centre and COVID-19 unit to provide treatment to the COVID-19 patient. Besides, CPA is trying to provide an incentive to the frontline workers including pilots, tug master and crews to recognize their heroic contribution during the pandemic situation.

## Findings and recommendations

Port is an area where several stakeholders are involved in the port operation with different segments. In port operation, port authority and customs are the separate body purposing the export-import facilitation. Coordination and cooperation between these bodies are badly needed in the situation of uncertainty and emergency. The COVID-19 has numerous impacts on the whole shipping industry including the port. Almost all of the impacts of COVID-19 in Chittagong Port activities have already been discussed by our informants as well as within the literature review. The policymakers may consider the identified gaps for future preparations. Of course, not all the impacts could be avoidable but several of them could be avoided with the help of cumulative actions.

During the initial COVID period, CPA was halted due to container congestion. In this regard, facilitating container stuffing and unstuffing operation outside of port premises (preferably ICD) can give a flow in container operation. Besides, custom permits only limited types of import cargoes through ICD. Allowing all types of import cargoes through ICD can reduce container congestion significantly. Despite continuous efforts, there are still a lot of paper documentation requirements for both export and import operation. Process optimization with the help of atomization and procedural simplification could be the key to system optimization. Chittagong Port can optimize the vessel call procedures by introducing an online vessel call management system where the pre-berthing documentation, berthing meeting, vessel scheduling, loading and unloading schedules could be managed centrally. Besides, extended atomization can be introduced in port yard operations in all possible segments. These will accelerate the process as well as increase transparency on the port operation. Again, due to the limited operation of custom and bank in COVID, the importer could not complete the lengthy paper process in most cases and was unable to release cargo within the scheduled time. Furthermore, with the aid of IT-based custom activities and a port community system, the CPA can customize the procedures to further accelerate the efficiency in both export and import operation. Here, the proposed ‘National Single Window’ (NSW) can be an effective model. As per the project documentation, NSW described it as the single electronic gateway that permits traders to submit all import, export and transit documents required by customs and other key regulatory agencies instead of a complex paper-based procedure. Simultaneously, introducing an online bank payment system can quicken the process further. CPA is also facing a continuous shortage of cargo capacity which requires immediate attention. CPA should establish efficient coordination with transshipment ports (mainly ports of Singapore and Colombo) to reduce the container congestion on its premises. In the long run, CPA also has to accelerate all the ongoing and forthcoming development projects to prepare for the growing demands. On the other hand, CPA needs to decentralize their dependability on road networks which currently facilitating the majority of the cargoes. Inland waterways and rail networks should be improved to versatile the choice of transportation. Establishing ICD based on IWT and rail networks can attract the respective mode of transportation. Besides, human workforces are mostly affected in an emergency like a pandemic. CPA’s strategy of work shifting/rotation can be permanently taken into consideration as an alternative plan. Accordingly, the existing workforce can be distributed in several groups to create availability as a reserve team in any situation. Table 2 summarizes the findings and associate recommendations.

**Table 2:** Impact on port and port facilities

Impacted port area		Impacts	Recommendations
Maritime (water) area	Outer anchorage, inner anchorage, river channel	Vessel waiting period increased, turnaround time increased, vessel calls decreased	• Implementing online-based vessel call procedure • Development of new terminals • Cooperation with transshipment port
Main port (land) area	Port facilities, port yard (warehouse), customs	Shortage of workers, death of port employees, shortage of storage (warehouse overloaded), limited custom activities	• Distribution of existing workforce to create availability as reserve team at any situation • Extend automation in port operation • Facilitating stuffing and unstuffing service outside of port premises • Allowing direct shipment of all import cargoes at ICD • Implementation of IT-based customs activities and port community system • Accelerating ongoing port development
Hinterland (outer port) area	ICD, road, rail, and inland water transportation	Shortage of transport vehicles/facilities, factory paused, limited bank activities	• Prioritizing IWT and rail network • Establishment of new IWT and rail-based off dock • Introducing an online bank payment system

## Conclusion

Port is a point between sea and land that needs a free flow of cargo and services. Any disruption in cargo flow and service can hamper the overall supply chain. COVID-19 crisis resulted in similar consequences. It is not only liable to loss of life but also responsible for halting the overall economy following a major interruption in the country’s busiest seaport. This empirical study attempted to find out the impacts of COVID-19 on Chittagong Port and the steps taken by Chittagong Port to overcome the situation.

As this was the sudden onset of a pandemic, initially CPA could not assume the impact of the pandemic, but soon after the first phase, CPA took some well-suited decisions to come out from this unsuited situation. As per primary and secondary data, the first phase of the government holidays slowed down the overall operation. Although Chittagong Port never stopped its operations, pandemic uncertainty halted cargo release. As a result, port yard storage capacity became full with a record number of containers compelled to slow down the cargo discharging. Therefore, the vessel row for berthing became long at outer anchorage. Simultaneously, the vessel waiting period, turnaround time and dwell time shoot up rapidly. Besides the transport crisis, COVID-19 affected port workers. Limited customs and banking activities worsened the situation. However, the CPA came up with a time-bound solution to keep the port operational. These include the direct delivery of containers through off docks instead of port premises, human gathering reduction, safety items distribution, shifting work of port employees and establishing COVID-19 unit inside CPA hospital. All these initiatives railed round to come out from this uncertain situation.

This study also presents a clear picture of how the CPA should be preparing for any future disruption on port operations. In line with the global trend, the CPA has to introduce more and more atomization in all possible segments. Vessel call procedures need to be online. Chittagong is a unique port where the container stuffing and de-stuffing are placed on the dock, i.e. at the port area on CFS (container freight station) shed. After solving the existing legal and security barriers, container stuffing and de-stuffing service should be kept outside of port premises to reduce the container congestion inside port premises. IT-based port and customs services and the formation of a monitoring cell by trained port officials could accelerate this process. To ensure safer and cheaper hinterland connections, CPA should introduce extended use of water transportation (seafarer was on isolation but not the truck drivers) and may consider to extend present railroad and port link road. On the other hand, CPA should ensure timely implementation of the ongoing projects including Matarbari Port, bay terminal and Potenga container terminal to cope with the future requirement. Besides, to attend the future need, they should also consider more infrastructure and new port facility development including berths and jetties development.

## Appendices

### Appendix A: Research Questions

What are the impacts of Covid-19 on Vessel calls (Vessel Numbers/Waiting period) in Chittagong port?

What are the impacts of port call procedure (Extra delay)?

What is the reason behind the change in waiting period?

What are the impacts on warehouse and distribution activities (ICD, Terminals, and Sheds)?

What are the impacts on Hinterland transport?

What are the impacts on Port Workers Due to COVID -19?

What are the actions taken by Chittagong Port to overcome the impact of COVID-19?

What more could be done by Chittagong port to reduce the impacts of COVID-19?

### Appendix B: Details of the interviewee

**Table:**

Serial no.	Date of interview	Duration (minutes)	Organization	Sector/category	Work experience (years)
R1	15 June	20	CPA (marine dept.)	Policymaker	29
R2	15 June	30	CPA (traffic dept.)	Policymaker	20
R3	15 June	10	CPA (VTSS)	Govt. agency	5
R4	15 June	30	CPA (admin)	Govt. agency	20
R5	28 June	25	CPA (craft division)	Govt. agency	27
R6	2 July	30	CPA (marine dept.)	Policymaker	34
R7	2 July	30	CPA (planning division)	Policymaker	25
R8	4 July	20	CPA (marine dept.)	Govt. agency	23
R9	5 July	50	Academic institution	Researcher	30
R10	13 July	40	Port agent	Port user	12
R11	15 July	30	Port agent	Port user	25
R12	25 July	12	Shipping agent association	Businessman	20
R13	28 July	15	Private ICD	Entrepreneur	10
